# Flow diagram of the differential diagnosis and clinical decision making in a rare case of contrast-induced encephalopathy following cardiac catheterization: a case report

**DOI:** 10.1186/s12872-023-03288-7

**Published:** 2023-06-01

**Authors:** Jiayin Sun, Lichuang Yuan, Hailiang Yu, Yanzhao Yang, Zhiming Zhou, Dean Jia, Yujie Zhou, Shiwei Yang

**Affiliations:** 1 Department of Cardiology, Beijing Anzhen Hospital, Capital Medical University, Beijing Institute of Heart, Lung and Blood Vessel Disease, The Key Laboratory of Remodeling-Related Cardiovascular Disease, Ministry of Education, Beijing, China; 2 Department of Cardiology, Beijing Hepingli Hospital, Beijing, China; 3 Department of Cardiology, Tangshan Fengrun District Second People’s Hospital, Tangshan, Hebei Province China; 4 Department of Cardiology, The First Hospital of Fangshan District, Beijing, China

**Keywords:** Contrast-induced encephalopathy, Cardiac catheterization, Differential diagnosis, Flow diagram, Case report

## Abstract

**Background:**

Contrast-induced encephalopathy (CIE) is considered as an uncommon complication following cardiac catheterization. Due to the varied manifestations, CIE has no formal diagnostic criteria. In fact, the incidence of CIE may be greatly underestimated because of the difficulty in its differential diagnosis with other cerebrovascular complications. Thus, making a flow diagram according to patients’ clinical symptoms and examinations after cardiac catheterization to help clinicians diagnose CIE is important and needed.

**Case presentation:**

In this report, we describe a case of probable CIE in a 66-year-old Chinese man with hypertension who underwent cardiac catheterization with stents placement in the bifurcation lesion, during which 80 ml iopromide contrast was used. About 2 h following the procedure, the patient lost his consciousness suddenly and suffered from a status epilepticus. Malignant arrhythmias were not found through continuous electrocardiogram monitoring, but mild ST-segment elevation was displayed in leads I and aVL. The echocardiography, plasma glucose and electrolyte levels were normal. Emergency re-angiography with percutaneous transluminal coronary angioplasty was performed in the culprit lesion, which involved 60 ml iopromide contrast. However, the patient remained unconsciousness and epilepticus. Non-contrast computed tomography (CT) of the head showed cortical and subarachnoid enhancement as well as prolonged retention of contrast media in the middle cerebral artery. With supportive treatment of intravenous hydration, sedative and dehydrant, the patient recovered 3 h later and finally discharged without any neurological deficits.

**Conclusions:**

CIE is an acute reversible encephalopathy induced by contrast media. It is exceptionally challenging to make the diagnosis of CIE following cardiac catheterization since there is a lack of consensus on the definition of CIE. Via this case we reviewed the related literatures, through which a flow diagram of the differential diagnosis and clinical decision making was given, which could help to differentiate CIE from other neurological complications following cardiac catheterization.

**Supplementary Information:**

The online version contains supplementary material available at 10.1186/s12872-023-03288-7.

## Background

Contrast media is an important diagnostic tool which is widely used in many imaging methods [1]. However, the application of contrast media could also induce some side effects, including hypersensitivity reactions [2], nephropathy [3], heart failure [4], arrhythmia [4] and encephalopathy [5–8]. Contrast-induced encephalopathy (CIE), mostly occurred in the invasive cerebrovascular procedure, is considered as an uncommon complication following cardiac catheterization [5–8]. There have been only a few dozen cases since the first case of CIE was reported in 1970 [9]. In fact, the incidence of CIE may be greatly underestimated because of the difficulty in its differential diagnosis with other hemorrhagic and thromboembolic cerebrovascular complications. Due to the heterogeneity of clinical manifestations, imaging evidence and reversible course are of great value in diagnosis and differential diagnosis [6, 10]. Here, we discuss a case of probable CIE in a 66-year-old man without any history of neurologic illnesses who lost consciousness and suffered seizures after cardiac catheterization. Via this case, we present a flow diagram of the differential diagnosis and clinical decision making in patients appeared neurological symptoms following cardiac catheterization.

## Case presentation

**Fig. 1:**
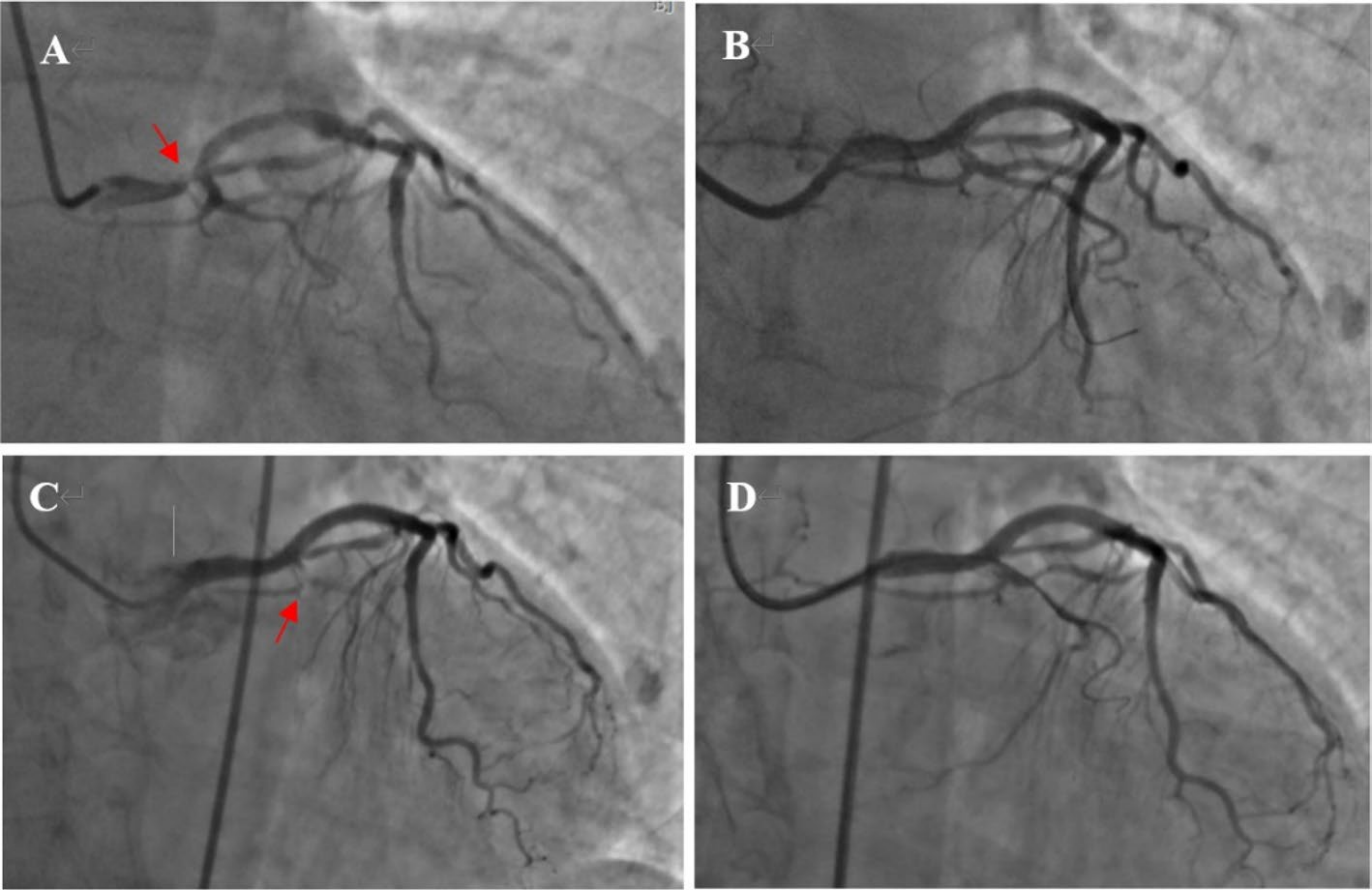
First intervention and emergency intervention. **a** Coronary angiography demonstrated a bifurcation lesion in LM, LAD and LCX (red arrow). **b** No significant residual stenosis was observed from LM to LAD after LM-LAD drug-eluting stent implantation. **c** Emergency re-angiography revealed TIMI 1–2 blood flow in LCX (red arrow). **d** TIMI 2–3 blood flow was restored in LCX after percutaneous transluminal coronary angioplasty. LM, left main. LAD, left anterior descending. LCX, left circumflex

**Fig. 2:**
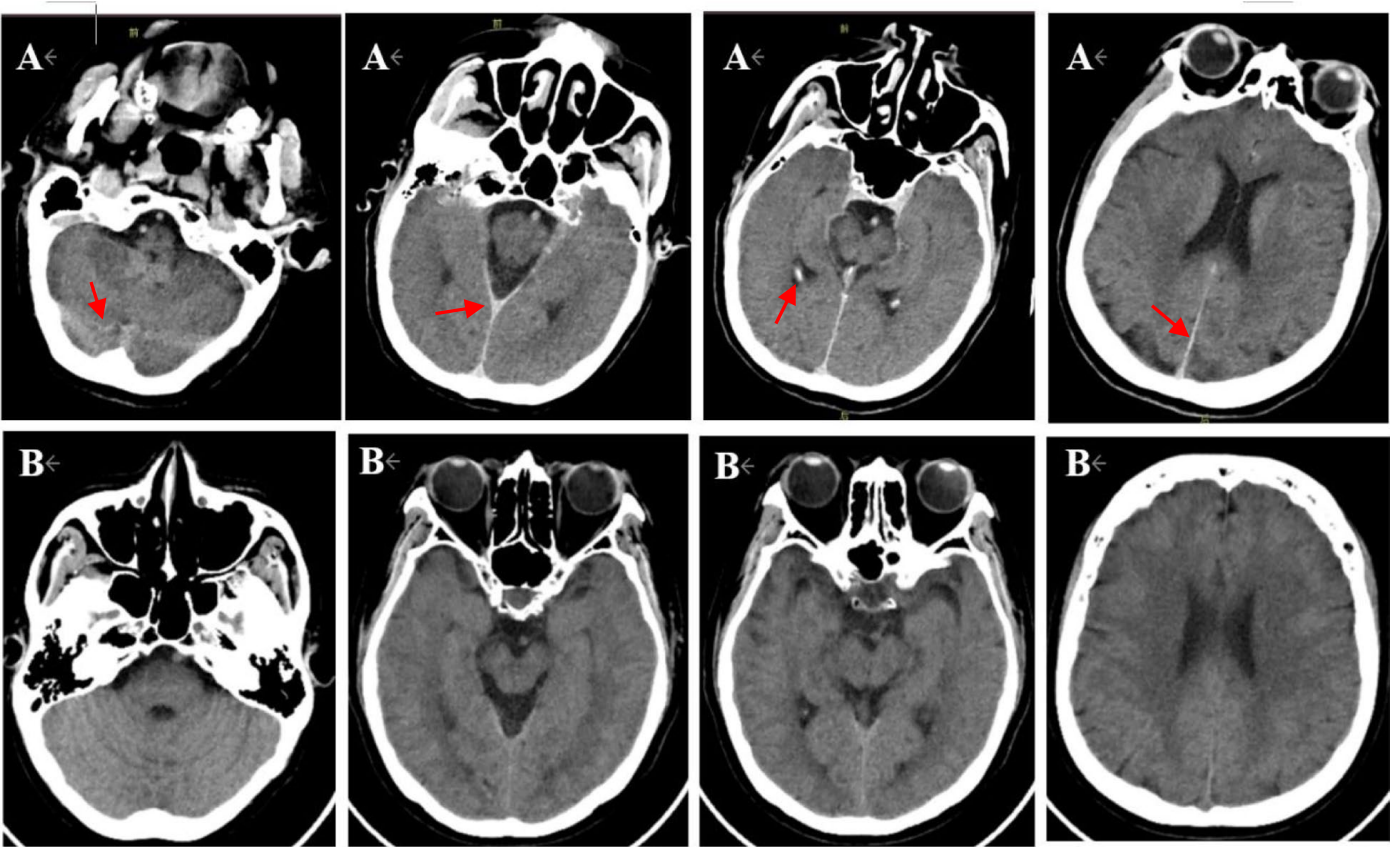
First and second non-contrast CT scans of brain. **a** Typical cortical and subarachnoid enhancement as well as prolonged retention of contrast media in the middle cerebral artery were observed in non-contrast CT scan (red arrow). **b** Non-contrast CT scan 36 h later showed resolution of the cortical and subarachnoid enhancement

A 66-year-old Chinese male patient with a history of hypertension for more than 20 years was admitted to hospital due to unstable angina. He didn’t have any history of neurologic illnesses or allergies, as well as any other heart disease besides coronary artery disease. In addition, he had a family history of hypertension. Physical examination on admission showed that his heart rate was 62 beats per minute, blood pressure was 130/80 mmHg, body mass index was 21.13 kg/m^2^ and the other findings were normal. A 12-lead Electrocardiogram (ECG) demonstrated sinus rhythm, with no ischemic ST/T abnormalities. Troponin I, B-type natriuretic peptide (BNP), blood glucose as well as lipid panel were within normal limits. The patient’s baseline estimated glomerular filtration rate (eGFR) was 93 mL/min/BSAc. The coronary angiography demonstrated a bifurcation lesion in left main (LM), left anterior descending (LAD) and left circumflex (LCX) coronary artery (Fig. 1a). The jailed balloon technique was used to prevent acute LCX occlusion at the time of LM-LAD drug-eluting stents implantation. After the procedure, no significant residual stenosis was observed from LM to LAD and TIMI 3 blood flow was acquired in LCX (Fig. 1b). The procedure lasted fifty-four minutes, and a total of 80 ml of non-ionic, low-osmolar, iodine-based contrast media (iopromide; Ultravist 370, Bayer Healthcare) was used. The patient returned to the ward with clear consciousness and no chest pain. Physical examination showed the heart rate was 74 beats per minute, blood pressure was 161/89 mmHg. A 12-lead ECG revealed the height of T wave was a little increased in leads I and aVL compared with the previous ECG, and the patient was given continuous ECG monitoring and tirofiban pumping. About 2 h after the procedure, the patient lost his consciousness suddenly and suffered from a status epilepticus. His bilateral pupils were generally equal in size and round, and the light reflection was sensitive. Bilateral Babinski and Chaddock signs were positive, but no neck stiffness was observed. His Glasgow Coma Scale was 8/15. A 12-lead ECG showed mild ST-segment elevation in leads I and aVL, together with ST-segment depression and T wave inversion in leads II, III and aVF. Continuous ECG monitoring did not find any malignant arrhythmias. And echocardiography confirmed no cardiac tamponade. His plasma glucose and electrolyte levels were maintained normal. Midazolam (10 mg, i.v.) was used to the patient for keeping sedation. Then, emergency re-angiography revealed TIMI 1–2 blood flow in LCX (Fig. 1c). Percutaneous transluminal coronary angioplasty (PTCA) was performed immediately and the TIMI 2–3 blood flow was restored (Fig. 1d). About 60 ml iopromide was utilized this time. A 12-lead ECG after PTCA revealed a significant decrease in elevated ST-segment in leads I and aVL. The patient was still in the state of status epilepticus and unconsciousness. Emergency non-contrast brain CT examination 3 h after first surgery was subsequently performed to exclude intracranial hemorrhage or ischemic stroke and revealed no obvious abnormalities. However, typical cortical and subarachnoid enhancement as well as prolonged retention of contrast media in the middle cerebral artery were observed one hour later after PTCA (Fig. 2a). Therefore, CIE was suspected, and the patient was given intravenous hydration to accelerate contrast media excretion, midazolam 1 mg per hour to treat epilepsy, mannitol 125 mL to dehydrate and reduce intracranial pressure. About 3 h later (6 h after the first surgery), the patient suddenly regained his consciousness, the seizures ceased, and all pathological normalized. Repeat non-contrast CT-brain was conducted 42 h after the first surgery, which showed resolution of the cortical and subarachnoid enhancement (Fig. 2b). Furthermore, there was no residual retention of contrast media in the middle cerebral artery. During the following 6 months, the patient did not experience any neurological symptoms.

## Discussion

CIE is a rare adverse effect taking place after procedures applying iodinated contrast media, which is predominantly associated with endovascular intervention or diagnostic imaging directly involving the cerebral circulation [11–14], and relatively less common during coronary angiography [15–17]. Different types (high-/ low-osmolality, ionic or nonionic) and dosages of contrast media can lead to CIE in patients with or without cardiovascular risk factors [5–27]. CIE has received less attention than other complications caused by contrast media [5–27]. Furthermore, there is a lack of consensus on the definition of CIE. Currently, the understanding of CIE is that it is an acute reversible encephalopathy induced by contrast media and occurred within minutes to hours after the procedure. The risk factors may include advanced age, male sex, hypertension, renal failure, impaired cerebral autoregulation and transient ischemic attack, but no single factor can explain all cases [7, 16]. In addition, the exact pathophysiology of CIE remains undetermined. Osmotic disruption of the blood–brain barrier, resulting in cerebral edema and neurologic dysfunction is considered to be the likely mechanism, while arterial vasospasm and disruption of the microcirculation of an already impaired cerebral auto-regulatory system is also seemed to lead to this condition [19]. Our patient, a 66-year-old man had a history of hypertension, which may impair cerebral autoregulation and increase the risk of CIE.

**Fig. 3:**
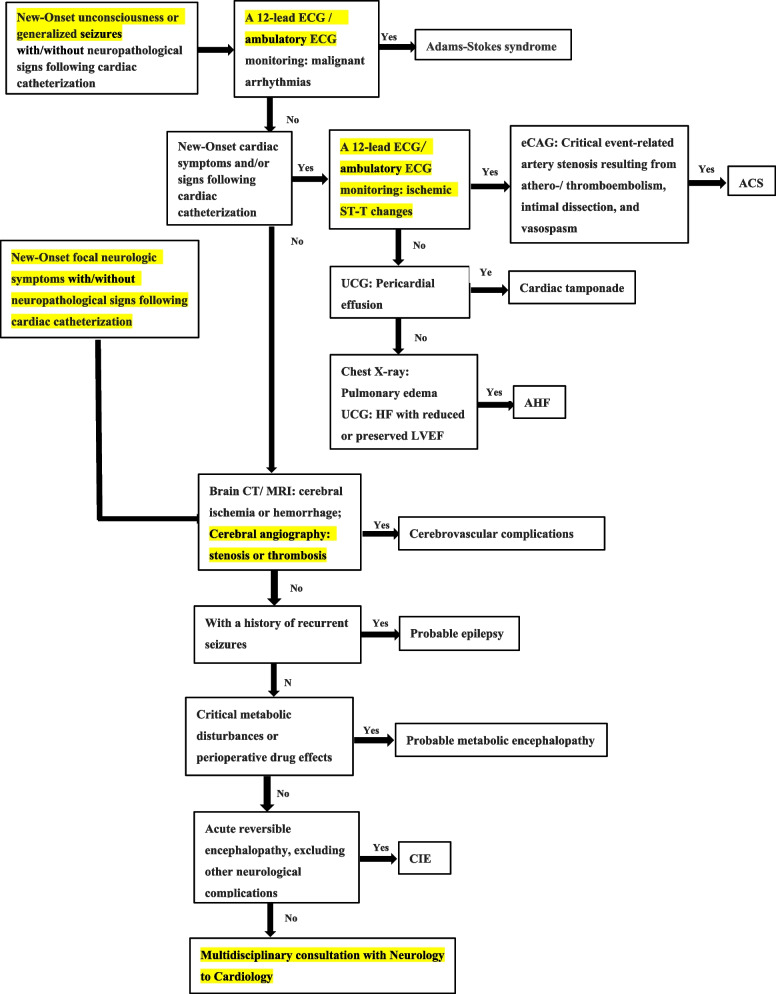
Flow diagram of differential diagnosis and clinical decision making of contrast-induced encephalopathy following cardiac catheterization. ECG: electrocardiogram; eCAG: emergency coronary angiography; ACS: acute coronary syndrome; UCG: ultrasonic cardiogram; HF: heart failure; LVEF: left ventricular ejection fraction; AHF: acute heart failure; CT: computed tomography; MRI: magnetic resonance imagining; CIE: contrast-induced encephalopathy

Since CIE appears to be in a critical condition after rapid onset, it is challenging to make the diagnosis of CIE prospectively and exclude other neurological complications following cardiac catheterization, including hypoxic-ischemic encephalopathy (HIE) after malignant arrhythmia, hemorrhagic and ischemic stroke, epilepsy, metabolic abnormalities and drug effects [6, 10]. With regards to symptomatology of CIE, transient cortical blindness is the most common manifestation. In addition, other heterogenic symptoms are all reported in previous cases, including focal neurological deficits such as visual disturbances, aphasia, motor and sensory deficits, as well as generalized syndromes such as loss of consciousness and seizures [6, 10]. Neuropathological signs may or may not appear in CIE, which are lack of specific differential diagnostic value. However, detailed past history such as cerebral vascular disease, epilepsy, cardiac arrhythmia, metabolic disease and allergy, is very important in the diagnosis and differential diagnosis when neurologic symptoms appeared following cardiac catheterization. Furthermore, if patients lose consciousness and/or suffer from seizures after cardiac angiography or intervention, cardiogenic shock should be firstly considered. In this condition, a 12-lead ECG and ambulatory ECG monitoring play an important role in differentiating Adams-Stokes syndrome caused by malignant arrhythmia. If new ischemic ST/T changes occurred in ECG, acute coronary syndrome is indicated and emergency coronary angiography should be conducted to evaluate coronary arteries. Moreover, ultrasonic cardiogram and chest X-ray are crucial in differentiating cardiac tamponade as well as acute heart failure. In case unconsciousness and seizures can’t be explained by hypoxic-ischemic encephalopathy following cardiogenic shock, cerebrovascular complications including cerebral ischemia and hemorrhage, which could be identified by brain CT or magnetic resonance imaging (MRI), should be considered then. In few patients with difficulty in differential diagnosis with brain CT or MRI, cerebral angiography can adequately exclude hemorrhagic and ischemic stroke. Patients of epilepsy usually have a history of recurrent seizures. Although metabolic encephalopathy has a low incidence, it should also be thought about in patients with critical metabolic disturbances. Additionally, for patients developing new-onset focal neurologic symptoms, cerebrovascular complications are the most common cause and cerebral imaging is preferred (Fig. 3).

It has been reported that majority of CIE patients have a good prognosis with neuropathological symptoms, signs and neuroimaging findings complete resolution within 48–72 h [6, 18–20]. Thus, if other common neurological complications have been excluded and symptoms of neurological dysfunction fully recover within 72 h, CIE should be taken into account. In addition, although neuroimaging findings in CIE may be normal or mimic cerebral hemorrhage or ischemia, they help in the differential diagnosis. A characteristic finding on non-contrast CT scan of the brain is cortical or subcortical contrast enhancement, with or without cerebrovascular contrast agent retention in corresponding areas [14, 15]. Characteristic findings on MRI of the brain consist of hyperintense signals on T2, DWI and FLAIR modalities in the affected regions.

Due to the low prevalence and varied manifestation, CIE has no formal diagnostic criteria, so does treatment protocol. However, the disorder is self-limiting, treatment is mainly supportive therapy, consisting of intravenous hydration and close observation. Other therapeutic agents used include anticonvulsants, mannitol, corticosteroids, and diuretics, which is focused on complications of CIE rather than the disorder itself [5, 6, 10–27].

## Conclusion

CIE is an acute and reversible neurological disorder with a variety of manifestations, which is rare following cardiac catheterization. Since there is a lack of consensus on the definition of CIE, its diagnosis should exclude other complications which cause neurological symptoms. For patients suffering from unconsciousness and/or seizures, HIE following cardiogenic shock as well as hemorrhagic and ischemic stroke, epilepsy and metabolic encephalopathy should be differentiated before CIE diagnosis. Fore patients developing new-onset focal neurologic symptoms, CIE must be considered in differential diagnosis of cerebrovascular complications following cardiac catheterization. In this report, we presented a rare case of typical CIE and reviewed the related literature, through which we drew a flow diagram of the differential diagnosis and clinical decision making, which could help to differentiate CIE from other neurological complications following cardiac catheterization.

## Supplementary Information

A**dditional file 1.** Timeline.

## Data Availability

The datasets used and/or analyzed during the current study available from the corresponding author on reasonable request.

## Abbreviations

CIE: Contrast-induced encephalopathy
LM: Left main
LAD: Left anterior descending
LCX: Left circumflex
ECG: Electrocardiogram
PTCA: Percutaneous transluminal coronary angioplasty
HIE: Hypoxic-ischemic encephalopathy
MRI: Magnetic resonance imaging
