# Supplementary material for: Flow diagram of the differential diagnosis and clinical decision making in a rare case of contrast-induced encephalopathy following cardiac catheterization: a case report
Source: BMC Cardiovasc Disord. 2023 Jun 1;23:280. doi: 10.1186/s12872-023-03288-7 (PMC10233977; doi:10.1186/s12872-023-03288-7)
Supplement: Supplementary file 1 — Additional file 1. Timeline. [file 12872_2023_3288_MOESM1_ESM.docx]

**Timeline**

| Before cardiac catheterization |  | - A 66-year-old male patient was admitted to hospital due to unstable angina without any history of neurologic illnesses or allergies. |
| --- | --- | --- |
| During cardiac catheterization |  | - A bifurcation lesion in left main (LM), left anterior descending (LAD) and left circumflex (LCX) coronary artery was showed in the coronary angiography. (Figure1a) - Drug-eluting stents were implanted in the LM-LAD lesion, and blood flow of LCX was normal. (Figure 1b) - A total of 80 ml iopromide was used. |
| After cardiac catheterization | 2 hours | - The patient lost his consciousness suddenly and suffered from a status epilepticus. - Continuous ECG monitoring did not find any malignant arrhythmias, but a 12-lead ECG showed mild ST-segment elevation in leads I and aVL. The echocardiography, plasma glucose and electrolyte levels were normal. - Emergency re-angiography with percutaneous transluminal coronary angioplasty was performed in the LCX lesion (Figure 1c-1d), and 60ml iopromide contrast was used. |
|  | 3 hours | - **ST-segment** elevation was resolved, the patient remained unconscious and in a status epilepticus. - Non-contrast CT scan showed cortical and subarachnoid enhancement as well as prolonged retention of contrast media in the middle cerebral artery. (Figure 2a) |
|  | 6 hours | - The patient suddenly regained his consciousness and the seizures ceased. |
|  | 42 hours | - Repeat non-contrast CT-brain showed resolution of the cortical and subarachnoid enhancement. (Figure 2b) |
| Follow up (6 months) |  | - The patient had no abnormalities in nervous system. |
